# Comprehensive supervised heavy training program versus home training regimen in patients with subacromial impingement syndrome: a randomized trial

**DOI:** 10.1186/s12891-021-04969-0

**Published:** 2022-01-15

**Authors:** Pierre Schydlowsky, Marcin Szkudlarek, Ole Rintek Madsen

**Affiliations:** 1 Reumatologiklinikken i Værløse, Bymidten 11,1, 3500 Værløse, Denmark; 2 Department of Rheumatology, Zealand University Hospital at Køge, Køge, Denmark; 3 Institute of Clinical Medicine, University of Copenhagen, Copenhagen, Denmark; 4 Department of Rheumatology and Spine Diseases, Gentofte University Hospital and Rigshospitalet, DK-2900 Hellerup, Denmark

**Keywords:** Shoulder, Rotator cuff, Subacromial impingement syndrome, Training, Heavy slow resistance training

## Abstract

**Background:**

There is no consensus on the best training regimen for subacromial impingement syndrome (SIS). Several have been suggested, but never tested.

The purpose of the study is to compare a comprehensive supervised training regimen (STR) based on latest evidence including heavy slow resistance training with a validated home-based regimen (HTR). We hypothesized that the STR would be superior to the HTR.

**Methods:**

Randomised control trial with blinded assessor. 126 consecutive patients with SIS were recruited and equally randomised to 12 weeks of either supervised training regimen (STR), or home-based training regimen (HTR). Primary outcomes were Constant Score (CS) and Shoulder Rating Questionnaire (SRQ) from baseline and 6 months after completed training. Results were analyzed according to intention-to treat principles. The study was retrospectively registered in ClinicalTrials.gov. Date of registration: 07/06/2021. Identification number: NCT04915430.

**Results:**

CS improved by 22.7 points for the STR group and by 23,7 points for the HTR (*p* = 0.0001). The SRQ improved by 17.7 and 18.1 points for the STR and the HTR groups respectively (p = 0.0001). The inter-group changes were non-significant. All secondary outcomes (passive and active range of motion, pain on impingement test, and resisted muscle tests) improved in both groups, without significant inter-group difference.

**Conclusion:**

We found no significant difference between a comprehensive supervised training regimen including heavy training principles, and a home-based training program in patients with SIS.

## Background

Subacromial impingement syndrome (SIS) is the most common cause of shoulder pain. According to the definition adopted, it explains 30 to 86% of all cases with shoulder pain [1, 2]. The condition affects the rotator cuff tendons, especially the supraspinatus tendon [3]. Exercise therapy has been shown to reduce pain and improve function [4–8].

Even though most training regimen include strengthening exercises to the rotatorcuff and the scapula, there is still no consensus on which training regimen to recommend, neither regarding the type, number, and intensity of exercises, nor their duration and frequency. In a newly systematic review and metaanalysis, supervised training and self training resulted in equal improvement of pain and function, and larger improvement than no training for patients with subacromial pain syndrome. The included studies used various exercises and dosages, but all included elements of rotatorcuff and scapular muscles strengthening [6].

In another systematic review and meta-analysis of patients with SIS, eccentric exercises provided no improvement of function compared with other exercises. The exercise regimens showed both similarities and differences, emphasizing the disparity of conceptions as regards to training. All interventions focused on at least one of these two exercises: shoulder external rotation with the shoulder in neutral position, and shoulder abduction in the scapular plane with the thumb pointing up. There was no consensus on the duration of training, which ranged from 4 till 12 weeks, nor on the frequency or training, which varied between 2 times per week to 2 times per day. Painful exercises showed no significant differences compared to pain-free exercises, and training regimens of 6–8 weeks were almost as effective as 12 weeks training [8].

Based on a systematic review, Kuhn [9] has suggested an exercise regimen including a combination of motion, stretching, and strengthening of the rotator cuff and of the scapular muscles. This regimen has not been tested, but several studies support this approach [10–19]. Yet, to this day, there is no consensus on which training regimen to recommend.

Training programs based on strengthening eccentric exercises for the rotator cuff and strengthening concentric/eccentric exercises for the scapula stabilizers may prevent surgery and yield year-long lasting effects [10, 11]. Recent studies have emphasized the importance of including infraspinatus, trapezius inferior and serratus anterior muscles in the rehabilitation of SIS [10, 12–14].

Stretching of the shoulder can improve range of motion and function, and prevent muscle strain [15, 16], and stretching of the pectoralis minor and of the posterior capsule as well as proprioceptive training are often suggested [13, 14, 17], even though the effects of pectoralis minor stretching only have been documented in scapular kinematics outcomes. There may even be doubts about it’s clinical efficiency, as Gutiérrez-Espinoza et al. found no short term benefit of additional pectoralis minor stretching on a specific exercise program, in terms of functional improvement or pain reduction in patients with subacromial pain syndrome [20]. Those findings have to be confirmed though.

Correction of posterior shoulder tightness has been documented in patients with internal impingement syndrome [13, 16]. Even though it isn’t as well documented in SIS, it makes sense to restore flexibility deficits, as they may lead to scapular malpositioning [13]. Besides, two recent studies suggest that posterior shoulder stretching may improve pain and function in patients with SIS [18, 19].

Lately, there has been focus on tendon training, and heavy slow resistance training (HSRT) has reduced pain, and yielded high patient satisfaction for the rehabilitation of Achilles and patellar tendinopathy [21]. Besides, only heavy load training compared to moderate training seems to maintain tendon mechanical properties in old age [22]. We therefore decided to incorporate heavy training as well, even though it had not been tested on SIS, finding it likely that rotatorcuff tendons would react positively, tendons in the upper extremity having same mechanic and physiologic properties as the ones in the lower extremity.

In this study, we hypothesized, that a supervised exercise protocol (STR) based on motion, stretching, and muscle and tendon strengthening with HSRT and focus on both scapula stabilizing muscles, and rotator cuff tendons, would be superior to a simpler home exercise program that resulted in higher function score, and shoulder satisfaction than the untreated control group [5]. For the STR, exercises that most effectively stimulate scapular and rotator cuff muscles according to electromyography studies and a systematic review were chosen [9, 23]. The purpose of the study was not to examine the effect of a single parameter but to compare two different training regimens as a whole.

## Methods

**Fig. 1:**
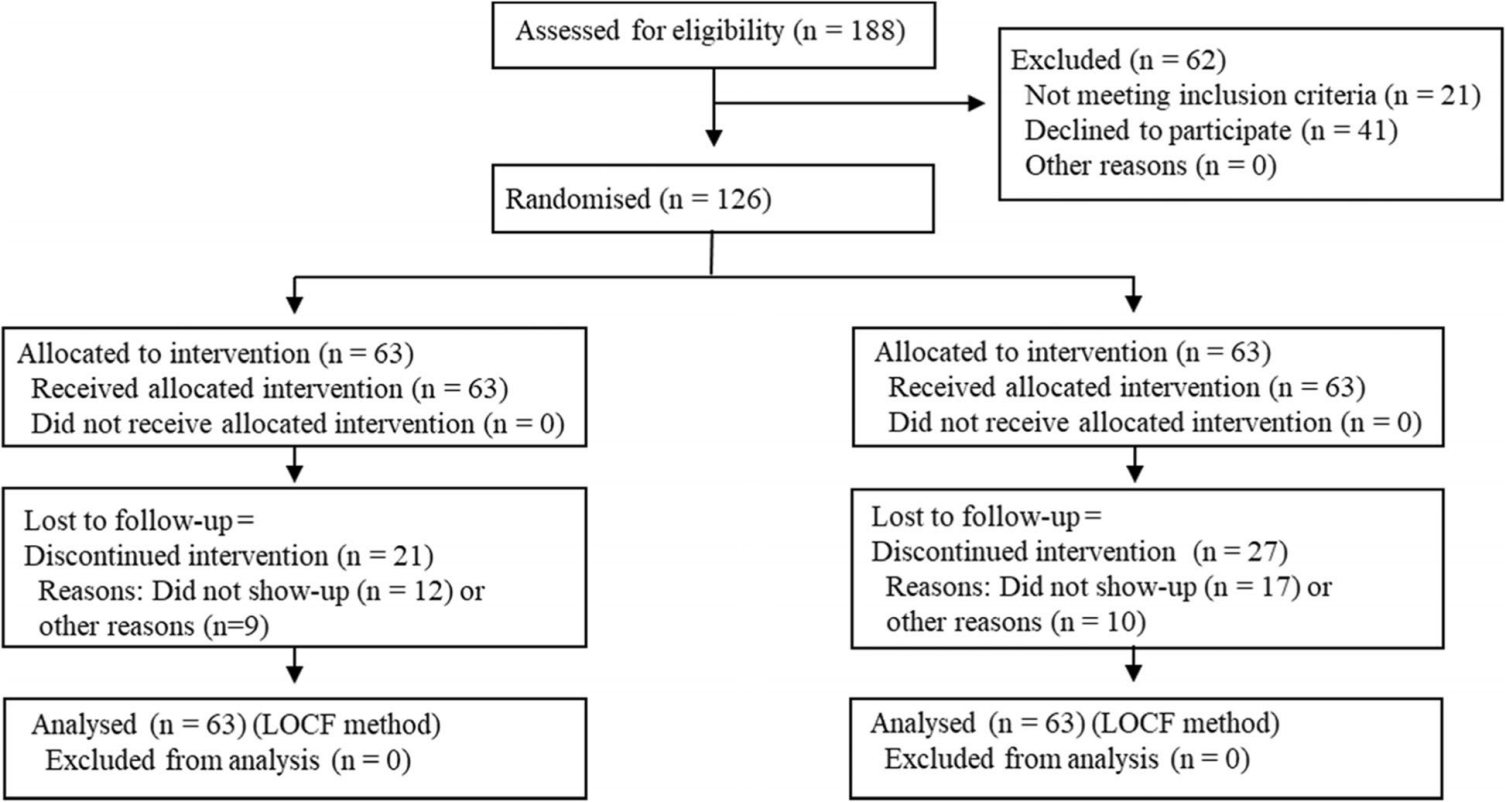
COMFORT flow diagram

In a randomised control trial, 188 successive patients referred to our clinic by their general practitioner from September 2013 to November 2017 were considered for participation. 126 patients fulfilled the inclusion criteria and agreed to participate. A CONSORT flow diagram is shown in Fig. 1. Allocation of the patients to either a supervised training regimen (STR) or a home training regimen (HTR) was concealed. All data were collected at our clinic.

Oral and written consent was obtained from all patients. Even though duration of symptoms wasn’t recorded, patients waited on average 4 months between time of referral from the General Practitioner and time of consultation in our clinic, due to the structure of the Danish system. There were thus no patients with duration of symptoms less than 4 months.

The 2 groups differed widely in terms of number of exercises and duration of training, the STR group having far more exercises and being more time-consuming than the HTR, the purpose of our study being to find out whether there was any benefit of the much more comprehensive STR program compared to the simpler HTR as a whole.

Training of participants in the STR group was supervised one-on-one by the same physiotherapist. Participants in the HTR group were provided with written instructions and shown how to perform exercises by the physiotherapist. They were assessed a week later to make sure that the exercises were performed correctly. No participant received any steroid injection during the trial.

The study was approved by the Ethics Committee of the Capital Region of Denmark on april 24th. 2013, protocol nr. H-4-2013-030.

### Inclusion criteria

- Subacromial impingement syndrome, regardless of age, sex, employment status, activity level, cause (trauma or overuse), and the severity and duration of symptoms. In order to fulfil inclusion patients had to reveal 3 positive tests out of 5: Positive Neer’s test, positive Hawkins test, positive Jobe’s test, painful arc, and pain on resisted external rotation of the arm [24]. All participants were examined with an ultrasound scanner at inclusion.

### Exclusion criteria

- Ongoing claim with the Labour market insurance, insurance company or comparable institution.
- Ongoing application for job revalidation or health related pension.
- Radiating neck pain.
- Ultrasound verified complete rotator cuff tendon lesion, as defined by hypoechoic or anechoic full thickness defect of the tendon, or absence of the tendon.
- Periarthritis humeroscapularis.
- Acute luxation or fracture of the shoulder.
- Ongoing steady analgetic treatment of other concomitant painful condition, unrelated to the patients shoulder problem.

### Flow

- All patients that fulfilled inclusion criteria, and did not have any exclusion criteria were offered participation in the trial. Age, sex, and self-reported employment status were recorded.
- All patients that accepted participation, were randomised blindly to either a supervised training regimen (STR) or a home training regimen (HTR).
- Participants in the STR group were supervised in the clinic.
- Participants in the HTR group were instructed in a home-based exercise regimen.
- All participants were examined with an ultrasound scanner at inclusion.

### Randomisation procedure

An equal amount of cards marked either group I (STR) or II (HTR) were folded and mixed, as to conceal the group for the examiner and the patient, who then picked up a card randomly.

### Training regimens

#### Supervised heavy training regimen (STR) [4, 9]

This training regimen consisted of 3 phases: the 1st focused on restoring motion, the 2nd on strengthening of the rotator cuff muscles, and the 3rd on strengthening of the scapular muscles. Stretching completed every training session.

- Motion training consisted of 6 exercises including postural and glenohumeral training.
  - Postural training consisted of shoulder shrugs and shoulder retraction exercises.
  - Glenohumeral training consisted of pendulum exercises, and active assisted flexion, abduction and external rotation. Motion training ended when the full passive flexion, abduction and external rotation had been re-established.
- Strengthening of the rotator cuff included 3 exercises: side lying external rotation, internal rotation and scaption.
- Strengthening of the scapular muscles included 5 exercises: press-up, push-up with a plus, serratus anterior supine punch, standing rowing (low row), and seated rowing (high row).
- Training sessions ended with 4 stretches: anterior shoulder stretch, posterior shoulder stretch, inferior capsular stretch, sleepers stretch.

Exercises were performed 3 times a week, with progression after the 1st week, and thereafter every 2nd week.

#### Choice of exercises

1. Motion exercises:

Our choice of motion exercises was based upon a systematic review, where Kuhn suggested that initial rehabilitation should include postural training with shoulder shrugs and shoulder retraction, pendulum exercises, and active assisted motion which have all been described and used in other studies [9, 25, 26]. Codman’s pendulum exercises are commonly prescribed after shoulder surgery and injury to provide distraction and oscillation resulting in decreased pain, increased flow of nutrients into the joint space, and early joint mobilization [27]. Some studies have questioned their relevance in SIS rehabilitation. Thus in a study that aimed at determining if added weight affected the shoulders ability to relax, Ellsworth et al. [27] showed that generally, the supraspinatus/upper trapezius muscle activity was significantly higher than the deltoid and infraspinatus activity - especially in the patients with pathological shoulders. The pendulum exercise with or without weight, did not have a significant effect on shoulder EMG activity though, neither in normal nor in pathological shoulders. And in another study on subjects having undergone subacromiel decompression [28], EMG analysis of the rotatorcuff showed that the supraspinatus tendon remained as passive during pendulum exercises as at resting baseline. It is therefore safe to assume that these exercises presented no risk in our population, but they may not be efficient for restoring passive shoulder range of movement, as a recent study on shoulder kinematics [29] has demonstrated that Codman pendulum exercises depend mainly on truncal movement and produce very little movement in the Glenohumeral and Scapulothoracic joints.

2. Strengthening exercises:

We combined a series of exercises, which value have been emphasized by several authors [5, 12, 13, 17, 23, 30–35].

- Side lying external rotation

The importance of including the external rotators in rehabilitation programs has been emphasized by several studies [12, 13, 30, 31].

Experimental shoulder pain elicited by injection of hypertonic saline in the supraspinatus muscle has an inhibitory effect on the activity of the infraspinatus muscle during arm elevation, but not on the activity of the scapulothoracic muscles. This indicate that it may be of importance to include strengthening of the infraspinatus in rotator cuff rehabilitation protocols [30]. Chaconas et al. [31] used a program basically consisting of standing resisted scapular retraction and posterior capsular stretch. Adding resisted external rotation was superior to active unresisted shoulder motion. We chose sidelying external rotation, as this exercise has a low Upper trapezius (UT)/Lower trapezius (LT) ratio, which is beneficial due to excessive UT activity and deficient LT activity in SIS [12, 13].

- Internal rotation

We included internal rotation training in order to ensure balance between internal and external rotators [32], as it has been speculated that weakness of the internal rotators may lead to shoulder pain [33].

- Scaption

Scaption stimulates the UT, but also the LT, the middle and lower serratus anterior (SA), the rhomboids, the levator scapulae and the supraspinatus [23]. The exercise probably offers the opportunity of training the supraspinatus with HSRT, and with the exception of the UT, stimulates the function of the scapular muscles, that is essential for shoulder recovery [12, 13, 17]. Besides, it makes sense to target the supraspinatus in rehabilitation programs, as it is the most frequently affected rotator cuff tendon [3]. In a small study on 11 patients waiting for subacromial decompression surgery, eccentric training of the supraspinatus muscle, resulted in 2 out of 4 patients cancelling their planned operation [36]^.^

- Press-up

This exercise puts focus on the pectoralis major muscle and the latissimus dorsi. These muscles generate power. Jobe and Pink suggested that they ought to be included as part of a comprehensive rehabilitation program, based upon stimulation of the shoulders protectors, positioners, pivoters, and propellers, which they named the 4P [34].

- Push-up with a plus

Push-up with a plus effectively stimulates the middle and lower serratus anterior, and the pectoralis minor muscle [23].

- Serratus anterior supine punch

The exercise has emphasis on the serratus anterior, whose decrease in activity has been linked to alterations in scapular and humeral motion during arm elevation [5].

- Standing rowing (low row)

Low row has been recommended as an exercise to stimulate scapular muscles without putting strain on the rotatorcuff [35]. Rowing effectively stimulates UT, MT, LT, rhomboids, and the levator scapulae [23].

- Sitted rowing (high row)

We included high rows to stimulate the upper part of the scapular stabilizing muscles in order to provide participants with a comprehensive rehabilitation program.

- Stretching exercises

Tightness of the pectoralis minor muscle, the posterior capsule and/or external rotators may lead to scapular dysfunction [37]. The unilateral pectoralis minor stretch is an efficient stretching method for this muscle [38] and may lead to less shoulder pain and improvement of function [39]. Stretching of the posterior shoulder structures with sleepers stretch and cross-body stretch can improve range of motion (ROM) and function [15, 16]. We added stretching of the inferior capsule to restore normal shoulder motion as well.

#### Home based exercise (HTR) [5]

This program has been validated [5]. The program consisted of the following:

- 1 motion exercise: upper trapezius relaxation
- 3 strengthening exercises: serratus anterior strengthening exercise, humeral external rotation with the arm at the side of the body, and humeral external rotation with a rubber band and the arm at 90 degrees abduction.
- 2 stretching exercises: posterior shoulder and pectoralis minor stretching,

Exercises were performed daily, with weekly progression.

Both the STR and the HTR groups exercised for 3 months and were encouraged to continue exercising on their own afterwards in case of persisting symptoms.

### Detailed training regimens

#### Supervised training regimen (STR)

- Motion training
- Postural training
- shoulder shrugs
- shoulder retraction
- Glenohumeral training
- Pendulum exercises
- Active assisted motion (with the help of the opposite arm, cane, or training cable)
- flexion
- abduction
- external rotation

Ad shoulder shrug and shoulder retraction: position is held for 10 s. Then pause for 10 s. Is repeated × 5.

Ad pendulum exercises: 20 small circular movements clockwise, 20 small circular movements anticlockwise, 20 small movements in the sagittal plane, and 20 small movements in the frontal plane.

Ad active assisted motion: the arm is slowly moved to the desired position and back again. Is repeated x 5.

Motion training ends when the full passive flexion, abduction and external rotation has been re-established.

- Strengthening of the rotatorcuff
- Side lying external rotation

The patient is lying on the opposite side. External rotation is performed with a hand weight or a rubber band

- Internal rotation

The patient is sitting or standing. Internal rotation is performed with a hand weight or a rubber band.

- Scaption

The patient is sitting or standing. The elbow is held in full extension, as the arm is lifted in the scapular plan, till 90 degrees elevation. Resistance is provided by hand weight or a rubber band.

- Strengthening of the scapular muscles
- Press-up

The patient is sitting on a chair, and grabs the side of the chair with each arm. The patient then lift the upper body from the chair.

- Push-up with a plus

The patient is lying prone on the floor. Push-ups are performed “with a plus” with protraction of the scapulas and hyper kyphosis of the thoracic spine, according to the description of Jobe et al. (26)

- Serratus anterior supine punch

The patient is lying supine on the floor or on a couch. A hand weight is held in each hand, and the arms are flexed 90 degrees, with the hand weights pointing toward the ceiling. The patient then lifts both shoulder blades up toward the ceiling.

- Standing rowing (low row)

Low rows are performed with the patient standing or sitting with the arms at 0 degree of flexion.

- Sitted rowing (high row)

The patient is sitting with the arms flexed 90 degrees, allowing high rows to be performed.

- Stretching
- Anterior shoulder stretch

The exercise is performed with the arm lifted up to 120 degrees of abduction, or lower if this position illicits pain.

- Posterior shoulder stretch

The arm is lifted up to 90 degrees, or lower if this position illicits pain. The opposite hand grabs the backside of the elbow and presses it towards the opposite shoulder.

- Inferior capsular stretch

The arm is lifted in full abduction. The opposite hand grabs the backside of the elbow and presses it towards the opposite shoulder.

- Sleepers stretch

The patient is lying on the painful side. The arm and elbow are flexed 90 degrees, the elbow pointing forwards. A gentle pressure downwards is applied on the wrist by the opposite hand.

Ad strengthening exercises (rotatorcuff and scapular muscles):

4 rows of exercises, with a decreasing number of repetition and increasing weight over time. Pause of 2–3 min between each rows. The exercises are performed slowly, with 3 s for the concentric fase, and 3 s for the eccentric fase (6 s per repetition), 3 times per week.

- 1st. week: 15 repetitions with maximal weight (RM)
- Week 2–3: 12 RM
- Week 4–5: 10 RM
- Week 6–8: 8 RM
- Week 9–12: 6 RM

Ad stretching exercises: the position is maintained for 60 seconds and repeated 4 times.

#### Home based exercise (HTR)

- Posterior shoulder stretch 30 s 5 times / day: the patients lifted their arm 90 degrees and pulled the elbow towards the opposite shoulder with their free hand.
- Pectoralis minor stretch 30 s 5 times / day: the patients placed each hand at shoulder height on adjacent walls of a corner and leant forward
- Upper trapezius relaxation exercise 5 times / day: the patients where instructed to abduct their arms without a shrugging the shoulders.
- Serratus anterior strengthening exercise: the patients lifted a weight vertically from a supine position and protraction of the scapulas.
- Humeral external rotation with a rubber band and the arm at the side of the body.
- Humeral external rotation with a rubber band and the arm at 90 degrees abduction.

Ad weight and rubber band training:

1. 10 repetitions × 3 per day the 1st. week.
2. 15 repetitioner × 3 per day the 2nd. week.
3. 20 repetitioner × 3 per day the 3rd. week.
4. On following weeks, the patients were instructed to repeat the repetition sessions with heavier weight, and rubber band with increased resistance.

Both the STR and the HTR groups exercised for 3 months, and were suggested to continue exercising on their own afterwards, in case of persisting symptoms.

### Compliance

We defined 3 levels of compliance.

- Level 1: full compliance, defined as performance of all planned training sessions.
- Level 2: partial compliance, where only part of the planned exercises sessions were performed.
- Level 3: no compliance.

Compliance was self-reported by the participants, and recorded at each visit.

### Evaluation

Data were only registered for patients included in the study. Assessment at entrance was performed by the main author, and all subsequent clinical evaluations by the same clinical assistant, blinded to the group the patient had been allocated to. The primary author made all the ultrasound exams at study entrance, prior to randomisation. Clinical assessment was performed at inclusion, at 4, 8, and 12 weeks after training start, and 6 months after ended training according to the protocol.

At each visit, we registered if training had been performed according to the instructions (full training, partial training, no training at all).

### Effect-variables

Primary effect variables

- CS (max. 100 points).
- SRQ (max 100 points).

For both CS and SRQ, higher scores indicated better outcome.

The CS assesses four aspects related to shoulder pathology; two subjective: pain and activities of daily living (ADL) and two objective: range of motion (ROM) and strength. The subjective components can receive up to 35 points and the objective 65, resulting in a possible maximum total score of 100 points (best function). Pain and ADL are answered by the patient; ROM and strength require a physical evaluation by the examiner. In a systematic review [40] quality ratings reached a level of 75% or higher. Studies evaluating the content validity of the Constant-Murley score suggest that the description in the original publication is insufficient to accomplish standardization between centers and evaluators. Despite this limitation, the Constant-Murley score correlates strongly (>or = 0.70) with shoulder-specific questionnaires, reaches acceptable benchmarks (rho > 0.80) for its reliability coefficients, and is responsive (effect sizes and standardized response mean > 0.80) for detecting improvement after intervention in a variety of shoulder pathologies.

The SRQ is a self-administered questionnaire designed to assess the severity of symptoms related to and the functional status of the shoulder. It includes domains of global assessment, pain, daily activities, recreational and athletic activities, work, satisfaction, and areas for improvement, some questions being rated more than others. It ranges between 17 and 100 (best score). It has been found to be valid, reliable, and responsive to clinical change [41].

Secondary effect variables

- Passive motion (flexion, abduction, internal and external rotation) was assessed with a goniometer.
- Active motion (flexion, abduction, internal and external rotation) was assessed with a goniometer.
- VAS (Visual Analogue Scale) on a 10 cm scale for each muscle test (full can test, empty can test, lift off test, resisted external rotation, palm-up test, Yergason’s test). 0 indicates no pain, and 10 worst pain.

### Full can test

The patient is seated or standing, holding their arm in 90° of elevation in the scapular plane and the patient’s thumb pointing up. The examiner then applies a downward force to the arm whilst the patient tries to resist it. The test is considered positive if the patient experiences pain in the arm.

### Empty can test

The patient is seated or standing, holding their arm in 90° of elevation in the scapular plane and the patient’s thumb pointing down. The examiner then applies a downward force to the arm whilst the patient tries to resist it. The test is considered positive if the patient experiences pain in the arm.

#### Lift off test

The patient is standing and is asked to place their hand behind their back with the dorsum of the hand resting in the region of the lumbar spine. The examiner then applies force against the patients hand whilst the patient tries to lift his hand off the back, increasing internal rotation. The test is considered positive if the patient experiences pain in the arm.

#### Resisted external rotation test

The patient is seated or standing with their arm by the side, and the elbow flexed at 90 degrees. The examiner stabilizes the elbow against the patients body with one hand. The patient is then asked to turn its forearm outwards, against the resistance of the examiners hand placed on the dorsal side of the wrist. The test is considered positive if the patient experiences pain in the arm.

#### Palm-up test

The patient is seated or standing. The palm-up test was performed with the arm elevated anteriorly against resistance while the elbow was in extension and the forearm supinated. The test is considered positive if the patient experiences pain in the anterior and upper part of the arm.

#### Yergason’s test

The patient is seated or standing. The patient’s elbow was flexed at 90 degrees with the arm by the side and the forearm in pronation. The patient was then asked to perform an active supination against the resistance of the physician’s hand placed on the wrist. The test is considered positive if the patient experiences pain in the bicipital groove.

- Neer’s test (rated positive = 1 and negative = 0).

The examiner stabilized the patient’s scapula with one hand, while passively flexing the patients arm with the other hand. If the patient reported pain in the arm in this position, the result of the test was considered to be positive

- Hawkins test (rated positive = 1 and negative = 0).

The patient’s arm was raised in front of the body to 90°, and then into internal rotation by the examiner. The test was positive if it elicited arm pain.

### Ultrasound

The integrity of the following structures was examined, and compared with the contralateral shoulder: caput longum bicipitis, the supraspinatus, infraspinatus and subscapularis tendons, and the sulcus bicipitalis and glenohumeral joint as regards to effusion. Tendons were evaluated as regards to thickening, as well as fibrillar disruption pointing at partial or full-thickness tear. Degeneration was defined as a tendon with a heterogenic ultrasound appearance, with loss of normal echogenic fibrillar appearance. These findings are associated with tendinopathy [42].

### Analysing strategy/statistics

Results were analyzed by an external statistician, according to intention to treat principles. The last observation carried forward (LOCF) method was applied for missing data from dropouts.

An a priori power analysis was performed based on the clinical important difference and on the standard deviation (SD) for CS. These two quantities vary considerable in the literature. Based on previous data [43–45] a clinically relevant between-group difference of 10 and a SD of 15 were chosen for analysis. A significance level of 5% and a power of 90% resulted in a total sample size of 100, i.e. 50 in each group. To minimize the risk of underpowering, a total of 126 patients were included in the study.

Descriptive statistics with mean and standard deviation were used to characterize the patients. The Student’s t-test was used to compare intra-group and inter-group results, and the Chi-squared test to compare binary outcomes.

The statistical software package IBM SPSS Statistics v. 25 was used for analyses.

## Results

63 participants were assigned to either the STR or the HTR group and analysed. The analysis was by original assigned groups.

The male/female ratio was 33/30 in the STR group and 32/31 in the HTR group.

The average age in the STR group was 61.7, and 60.3 years in group II (*p* = 0.60) (Table 1).

**Table 1:** Baseline characteristics

	STR Group	HTR Group	p
	*n* = 63	*n* = 63	
Age (mean ± SD)	61.7 ± 13.4	60.3 ± 13.0	0.56
Male/female (n)	33/30	32/31	–
Employment status:			
Employed/unemployed/full time sick leave/partial sick leave/retired (n)	33/2/2/0/26	33/3/2/0/25	–
**Shoulder scores (mean ± SD)**			
Constant Score (0–100)	37.7 ± 11.6	36.3 ± 9.7	0.47
Shoulder Rating Questionnaire (0–100)	54.1 ± 14.5	50.2 ± 14.9	0.14
**Motion (degrees)**			
Passive flexion	148.8 ± 37.8	141.6 ± 42.0	0.32
Passive abduction	119.7 ± 42.4	109.0 ± 40.1	0.15
Passive outer rotation	42.4 ± 11.6	43.1 ± 9.5	0.74
Passive inner rotation	3.0 ± 1.5	3.1 ± 1.6	0.61
Active flexion	145.1 ± 39.4	140.6 ± 42.4	0.54
Active abduction	115.0 ± 42.8	107.6 ± 40.3	0.32
Active outer rotation	42.4 ± 11.6	43.0 ± 9.5	0.77
Active inner rotation	3.0 ± 1.5	3.2 ± 1.6	0.50
**Muscle tests (VAS scale 0–10)**			
Full can test	4.4±2.7	5.4±2.9	0.04
Empty can test	4.7 ± 2.7	5.3 ± 2.9	0.19
Lift off test	3.4±3.3	4.4±3.3	0.12
Resisted external rotation test	3.1 ± 2.9	3.4 ± 2.9	0.61
Palm up test	5.1±3.3	5.0±3.1	0.95
Yergasons test	2.3 ± 2.4	2.0 ± 2.4	0.53
**Ultrasonography (n)**			
Joint effusion, yes/no	3/60	1/62	–
Sulcus effusion, yes/no	17/46	16/47	–
Normal/oedema/partial lesion/			
total lesion/degeneration (n)			
Caput longum bicipitis	56/7/0/0/0	53/8/1/1/0	–
Subscapularis	51/3/0/0/9	55/3/0/0/5	–
Supraspinatus	15/28/16/0/4	15/31/14/0/3	–
Infraspinatus	60/1/1/0/1	60/1/2/0/0	–

With the exception of Full can test, which had a higher VAS score at baseline in the HTR group (*p* = 0.04), both groups were identical as regards to all other parameters (Table 1).

### Intra group results (Tables 2 and 4)

- Within each group all parameters improved significantly between the 1st

**Table 2:** Changes in shoulder scores between visits

Variable	Visit	p	Visit	p	Visit	p	Visit	p	Visit	p	Visit	p
	4 vs. 1		4 vs. 1		5 vs. 1		5 vs. 1		5 vs. 4		5 vs. 4	
	STR Group		HTR Group		STR Group		HTR Group		STR Group		HTR Group	
	n = 63		n = 63		n = 63		n = 63		n = 63		n = 63	
	Mean ± SD		Mean ± SD		Mean ± SD		Mean ± SD		Mean ± SD		Mean ± SD	
**Shoulder scores**												
CS	16.0 ± 15.8	0.0001	19.7 ± 16.7	0.0001	22.7 ± 20.5	0.0001	23.7 ± 18.2	0.0001	6.7 ± 11.9	0.0001	4.0 ± 11.6	0.008
SRQ	11.7 ± 4.2	0.0001	16.4 ± 18.1	0.0001	17.7 ± 19.5	0.0001	18.1 ± 18.1	0.0001	6.0 ± 12.0	0.0001	1.6 ± 10.3	0.21
**Passive Motion (degrees)**												
Flexion	12.8 ± 35.7	0.0001	16.9 ± 38.3	0.0001	15.1 ± 38.6	0.038	21.4 ± 41.6	0.0001	2.26 ± 24.6	0.72	4.5 ± 22.0	0.09
Abduction	21.2 ± 38.1	0.0001	33.4 ± 44.4	0.0001	26.9 ± 39.4	0.003	39.3 ± 46.6	0.0001	5.9 ± 32.0	0.15	5.9 ± 31.7	0.14
Ext. rotation	4.6 ± 9.2	0.0001	3.2 ± 9.36	0.0001	5.2 ± 9.0	0.0001	3.8 ± 9.7	0.0020	0.65 ± 4.5	0.26	0.63 ± 2.9	0.10
Int. rotation	1.1 ± 1.5	0.0001	1.0 ± 1.5	0.0001	1.5 ± 1.6	0.0001	1.3 ± 1.8	0.0001	0.37 ± 1.1	0.01	0.30 ± 1.0	0.02
**Active Motion (degrees)**												
Flexion	13.9 ± 33.7	0.0001	17.7 ± 39.1	0.0001	16.5 ± 39.3	0.002	21.8 ± 42.7	0.0001	2.7 ± 26.9	0.44	4.06 ± 20.7	0.12
Abduction	24.1 ± 36.4	0.0001	34.0 ± 44.8	0.0001	30.0 ± 41.6	0.0001	39.2 ± 47.7	0.0001	5.9 ± 31.9	0.15	5.2 ± 31.5	0.19
Ext. rotation	4.6 ± 9.2	0.0001	3.3 ± 9.4	0.007	5.2 ± 9.0	0.0001	3.9 ± 9.7	0.002	0.65 ± 4.5	0.26	0.63 ± 2.9	0.09
Int. rotation	1.0 ± 1.5	0.0001	0.97 ± 1.6	0.0001	1.4 ± 1.6	0.0001	1.3 ± 1.9	0.0001	0.37 ± 1.1	0.01	0.30 ± 1.0	0.02
**Muscle tests (VAS scale)**												
Full can	−1.7 ± 2.7	0.0001	−1.9 ± 3.4	0.0001	−2.2 ± 3.0	0.0001	−1.9 ± 3.8	0.0001	−0.49 ± 2.0	0.06	−0.04 ± 2.5	0.90
Empty can	−1.9 ± 2.5	0.0001	−1.5 ± 2.8	0.0001	−1.9 ± 2.9	0.0001	−1.5 ± 3.2	0.0001	−0.02 ± 1.6	0.93	−0.04 ± 1.8	0.87
Lift off	−0.81 ± 2.7	0.02	−1.5 ± 3.4	0.0001	−1.0 ± 3.6	0.028	−1.7 ± 3.5	0.0001	−0.22 ± 2.4	0.46	−0.02 ± 1.5	0.30
Ext. rotation	−1.4 ± 2.4	0.0001	−1.40 ± 2.0	0.0001	−1.3 ± 2.7	0.0001	−1.6 ± 2.2	0.0001	0.07 ± 1.5	0.72	−0.20 ± 1.2	0.19
Palm up	−2.8 ± 3.0	0.0001	−2.3 ± 3.1	0.0001	−3.0 ± 3.1	0.0001	−2.5 ± 3.3	0.0001	−0.15 ± 1.9	0.51	−0.21 ± 1.9	0.39
Yergason	−0.92 ± 2,3	0.003	−0.72 ± 2.0	0.007	−0.90 ± 2.8	0.02	−0.81 ± 2	0.002	−0.09 ± 1,4	0.62	0.02 ± 1.3	0.90

and the 4th visit where patients had trained for 3 months and completed training, and between the 1st and the 5th visit, 6 months after training completion, during which participants were encouraged to continue training in case of persistent symptoms.

- Between the 1st. and 5th. visit, CS improved by 22.7 points for the STR group an

by 23.7 points for the HTR (*p* = 0.0001). The SRQ improved by 17.7 and 18.1 for the STR and the HTR groups respectively (p = 0.0001). Range of motions improved in all directions, best for passive and active abduction, which improved with 26.9 (*p* = 0.03) and 30 degrees for the STR group (*p* = 0.0001) and with respectively 39.3 and 39.2 degrees for the HTR group (p = 0.0001). Impingement tests normalised in both groups, best for the Neer’s test, which became negative in 60% of the cases in both training regimens (*p* < 0.0001). Hawkins test normalised by 23% for the STR group (*p* = 0.05) and 22% for the HTR group (*p* < 0.02). Resistive tests all achieved statistical significance with regard to diminished pain, best for the full can test, and the palm-up test. VAS scores improved respectively for the STR end the HTR groups by 2.2 and 1.9 for the full can test (p = 0.0001), and by 3.0 and 2.5 for the palm-up test (p = 0.0001).

- Between the 4th and the 5th visit, there was significant improvement in CS,

and both passive and active internal rotation in both groups. SRQ improved significantly for the STR group, but not for the HTR.

### Inter group results (Tables 3 and 4)

Neither between evaluation at inclusion and the 4th nor 5th visit, did we found any significant difference between the 2 groups, regardless of the variable considered.

**Table 3:** Inter-group differences (STR vs. HTR group) in score changes between visits

Variable	Inter-group	SE	p	Inter-group	SE	p	Inter-group	SE	p
	difference			difference			difference		
	Mean			Mean			Mean		
	Visit 4 vs. 1			Visit 5 vs. 1			Visit 5 vs. 4		
**Shoulder Scores**									
Constant score	−3.7	2.9	0.20	−1.0	3.5	0.77	2.7	2.1	0.20
SRQ	−4.8	2.9	0.10	−0.42	3.4	0.90	4.4	2.0	0.03
**Passive motion (degrees)**									
Flexion	−4.1	6.6	0.54	−6.3	7.16	0.38	−2.3	4.1	0.58
Abduction	−12.3	7.4	0.10	−12.4	7.7	0.11	−0.05	5.7	0.99
Ext. rotation	1.39	1.65	0.400	1.41	1.7	0.40	0.02	0.70	0.98
Int. rotation	0.03	0.27	0.90	0.11	0.31	0.73	0.07	0.19	0.70
**Active motion (degrees)**									
Flexion	−3.9	6.5	0.55	−5.3	7.3	0.47	−1.4	4.2	0.74
Abduction	−9.9	67.3	0.18	−9.2	8.0	0.25	0.65	5.6	0.91
Ext. rotation	1.32	1.6	0.43	1.34	1.7	0.42	0.02	1.7	0.98
Int. rotation	0.05	0.27	0.86	0.12	0.31	0.70	0.07	0.19	0.70
**Muscle tests (VAS scale)**									
Full can	0.23	0.55	0.67	−0.21	0.60	0.73	−0.45	0.41	0.28
Empty can	−0.36	0.48	0.45	−0.34	0.54	0.53	.02	0.30	0.95
Lift off	0.65	0.55	0.24	0.62	0.63	0.33	−0.03	0.35	0.93
Ext. rotation	0.00	0.39	0.99	0.27	0.43	0.54	0.27	0.24	0.26
Palm up	0.05	0.55	.878	−0.43	0.57	0.46	− 0.48	0.33	0.38
Yergason	−0.21	0.39	0.60	−0.09	0.43	0.83	0.11	0.24	0.65

*SE* standard error of difference

**Table 4:** Neer’s and Hawkins test for impingement results (positive or negative) with *p*-values for intra-group between-visits differences and inter-group differences

	STR Group (n = 63)						HTR Group (n = 63)						STR vs. HTR group		
	Visit						Visit						Visit		
	1	4	5	4 vs. 1	5 vs. 1	5 vs. 4	1	4	5	4 vs. 1	5 vs. 1	5 vs. 4	1 vs. 1	4 vs. 4	5 vs.5
	Positive:Negative			p			Positive:Negative			p			p		
	n:n						n:n								
Neer’s	51:11	17:45	14:48	< 0.0001	< 0.0001	0.55	56:8	21:43	18:46	< 0.0001	< 0.0001	0.45	0.41	0.51	0.48
Hawkins	41:21	37:25	30:32	0.34	0.11	0.10	45:19	33:31	31:33	< 0.03	< 0.008	0.88	0.10	0.34	1.00

Intra-group comparison of proportions was performed using McNemar’s test

Inter-group comparison of proportions was performed using the “N-1” Chi-squared test

The average improvements in CS and SRQ between visit 1 and 5 (and 4) may be considered not only statistically significant but also clinically meaningful in both groups although between group-differences were not observed [46]. This may be explained by spontaneous improvement and/or efficacy of both training regimens.

### Drop out rate (Table 5)

At visit 4, 13 patients had dropped out in the STR group and 19 in the HTR group (*p* = 0.2). The corresponding numbers for visit 5 were 21 and 27 (*p* = 0.3). Identifiable reasons for dropout were failure to perform the exercises because of pain, lack of time, or concomitant disease (Table 6).

**Table 5:** Dropouts

	STR Group	HTR Group	p
	*n* = 63	n = 63	
Dropout rate at			
Visit 2	5	8	0.418
Visit 3	8	12	0.335
Visit 4	13	19	0.218
Visit 5	21	27	0.274

“N-1” Chi-squared test as recommended by Campbell (2007) and Richardson (2011)

Campbell I (2007) Chi-squared and Fisher-Irwin tests of two-by-two tables with small sample recommendations. Statistics in Medicine 26:3661–3675. PubMed

Richardson JTE (2011) The analysis of 2 × 2 contingency tables - Yet again. Statistics in Medicine 30:890. PubMed

**Table 6:** Reasons for discontinuing the intervention (n)

Training group	STR	HTR
Non-compliant to training		2
Steroid injection	2	
New job	1	
Pain	1	3
Did not show up	12	17
Did not have the time	1	2
Preferred other kind of training		1
Hand surgery	1	
Moved	1	
Parkinsonism		1
Treatment with prednisolone	1	
Frozen shoulder	1	1
Total	21	27

### Compliance

We calculated a mean level of compliance, which was 1.38 for the STR group and 1.51 for the HTR group. The difference between the groups was non-significant (*p* = 0.093).

### Ultrasonography

No difference was found at baseline between the groups (Table 1).

## Discussion

Our study showed that the effect of a comprehensive training regimen including heavy training principles and consisting of 6 motion, 8 strengths and 4 stretching exercises performed under supervision of a physiotherapist was not better than a much simpler home-based program offering 1 motion, 3 progressive strength, and 2 stretching exercises. There was within-group improvement for almost all parameters, but no between-group differences.

### Supervised versus home-based training

The benefit of adding supervision to a training regimen including strengthening exercises is poorly documented. In a newly systematic review, supervised training and self training resulted in equal improvement of pain and function, and larger improvement than no training for patients with subacromial pain syndrome [6].

In another recent systematic review, supervised physical therapy and home-based progressive shoulder strengthening and stretching exercises for the rotatorcuff and scapular muscles were equally effective in patients with SIS treated conservatively [7].

It seems that supervision yields better compliance and more correct performance of the exercises [47–49]. However, in a study of patients who had undergone rotator cuff repair, no statistical differences were found between an exercise program under the supervision of a physiotherapist and a standardized home-based exercise program regarding pain, functional status, quality of life and depression status [50]. Both programs included active and strengthening exercises. Supervised rehabilitation with strengthening exercises of the rotator cuff and scapula stabilizers seems to be superior to home exercises focusing on mobility for improving shoulder function after arthroscopic acromioplasty [51]. Thus, the type of exercises offered seems to be important.

### The heavy training component

The poor result of the addition of heavy training, is in accordance with other studies [51–54].

Supervised strengthening program has been shown to be superior compared to home-based unresisted movement training [51], but high load training may not be superior to low load training. Thus, in a study of 100 patients, Ingwersen et al., found no difference between a group who trained according to the principles of Heavy Slow resistance training and a group who were rehabilitated with low level exercises [53]. In another study, Maenhout et al., added heavy eccentric training to a traditional rotator cuff strengthening program, resulting in higher isometric strength at 90 degrees of abduction, but without any effect on pain or function [54]. In a systematic review [52], it was found that for persistent subacromial pain, supervised and home-based strengthening leads to similar outcomes as surgery and that home-based heavy load eccentric training does not add benefits to home-based rotator cuff strengthening and physiotherapy.

HSRT has been shown to reduce pain in Achilles and patellar tendinopathy [21]. As training of the rotator cuff tendons is a substantial part of SAIS rehabilitation, it would therefore make sense, that HSRT would yield good results in that field as well. The results of our and other studies seem to contradict this assumption. It may be, that tendons involved in multidirectional actions, as for the rotator cuff, present different challenges to rehabilitation, than patellar and Achilles tendons, which mainly have a unidirectional function.

### The comprehensive regimen

Considering the complicated biomechanics of the shoulder, we were expecting that our program, consisting of a comprehensive set of exercises with focus on restoring mobility and strength of the scapular muscles and rotator cuff tendons, would reveal superior to the simpler program designed by Ludewig et al. [5]. But our population had an average age of over 60 years, and even though sport attendance wasn’t registered, we speculate that a younger and more sporty group, might have performed better in the STR group. This theory is supported by the observations of our physiotherapist, who found that in the supervised group, several patients had difficulties performing the exercises correctly. Unfortunately, this wasn’t assessed, but proper execution of instructed exercises has been reported as challenging, and in another Danish study, only a quarter of the patients performed their home-based shoulder abduction exercise correctly when evaluated 2 weeks after the instruction by a physiotherapist [47].

### Drop-out rate

Out of 48 patients lost to follow-up at visit 5, 12 from the STR group and 17 from the HTR group just didn’t show up, and 2 from the HTR group stopped doing the exercises. Other causes of dropout were failure to perform the exercises because of pain, lack of time, or concomitant disease. Participants were only assessed with ultrasonography of their shoulder at entrance. Any worsening was assessed clinically, and resulted in withdrawal from the study in a few cases. Two participants withdrew because they developed a frozen shoulder, and 6 because of pain. After withdrawal, ultrasonography was performed. In no cases did we detect any worsening of the rotatorcuff condition (unpublished data). We speculate that the duration of the trial, with the last follow-up being 9 months after inclusion might have been a substantial cause of drop-out. We were unfortunately unable to get in touch with the participants that didn’t show up, and can therefore not offer any other explanation.

### Compliance

Two important issues for the success of exercise therapy are correct performance of prescribed exercise and compliance, which is reported as variable, but often low, especially for home-based exercise regimens. Compliance for home based exercises was found to be as low as 29% in a Danish study on helicopter pilots [48] and as high as 86% in a study comparing eccentric with concentric supraspinatus training [36] even though supervised training regimens often have greater compliance than home based exercises [49]. In our study, contrary to our expectations, compliance defined as completion of the exercise regimens, was without significant difference between the two groups. We find it possible, that adherence to the training protocols, has been enhanced by the prospect of meeting up to assessment every month.

### Baseline characteristics

This was a randomised controlled trial. Consequently, the study groups were not “matched”. But it turned out that the two groups were comparable regarding most baseline characteristics shown in Table 1, with the exception of Full can test, which had a higher VAS score at baseline in group II (*p* = 0.04). We find it unlikely though, that this sole baseline characteristic would account for the lack of between-group difference in all end-result parameters. As this was a randomised controlled trial, spontaneous improvers are taken into account when comparing the groups.

### Study limitations/ possible flaws

The diagnosis of SAIS is a challenge [55–57]. Patients in the present study fulfilled criteria that are accepted as reliable and accurate [24]. Other papers investigating training regimen for rotator cuff elicited pain and function reduction may have operated with different definitions [53], making direct comparison difficult. Ultrasonography at entrance, revealed edema, partial lesions or pathological findings in the supraspinatus tendon in all but 15 participants in each group. These 15 participants may well have had a rotator cuff problem anyway, as ultrasonography, despite its usefulness in shoulder pathology, can show normal findings in patients with a clinical rotator syndrome [58]. We therefore believe, that the risk of having included patients with different pathologies is small.

Several patients refused to enter our project, due to intense shoulder pain. We did not include steroid injection as an exclusion criterion in this trial in order to mimic clinical daily practice as much as possible. When needed, due to pain, patients were therefore offered between 1 and 3 injections with approximately 4 weeks interval before they were re-invited to participate in the trial. This approach may have resulted in a biased population as patients who had experienced severe pain for a long time may have preferred standard physiotherapy instead of challenging exercises in a clinical trial. This possibility is emphasized by the fact that none of the patients that required more than 1 steroid injection accepted to enter the study (data not registered).

Recruitment of patients proved particularly difficult, which is why it took us about 5 years to complete the study. The reason is unclear as these patients were not characterized or registered. Severe pain, lack of time, transportation time etc. may have contributed. Consequently, the patient sample studied may not be completely representative for SAIS patients in general.

For obvious reason, neither the patients nor the physiotherapist were blinded to the exercise regimen. Most of the patients wished to participate in the STR and expressed disappointment when randomised to HTR. Theoretically, this could affect the results negatively in the control group, However, any significant difference in favor of STR failed to be shown.

### Consideration for future research – future exercise programs

One important consideration, when offering rehabilitation to a patient, is how to structure the program. In addition to having proper exercises, a rehabilitation program ought to promote good compliance with regards to correct exercise performance, amount of time spent on training in each training session and the duration of training over time. All of these training elements represent a challenge. Mobile Phone Text Messaging as Reminders of Home Exercises may be effective [59].

But low compliance may be due to other factors. In a Danish study, lower adherence to a 10-week exercise program of 2 or 12 min’ duration, performed 5 times a week, was predicted by poorer psychosocial work environment and lower exercise self-efficacy. Interestingly, a longer exercise program was not associated with lower adherence [60]. Future research ought to focus on how to motivate this group for better training compliance.

It seems probable, that a more tailored program could boost the effect of training. An approach to more individualised programs could include longer and more challenging programs for patients used to recreational or professional sport activities as these patients have high requirements for shoulder function and may find home-based exercise with focus on repetition and endurance tedious. Conversely, simpler home-based exercises might be offered to patients with low physical activity with no incentive to perform challenging programs.

New techniques may be added, to structure the best functional rehabilitation programs. Thus, in a study using 3D-measurement techniques, magnetic resonance images were coupled with shoulder during exercises. The study showed how the glenohumeral joint, the labrum and the subacromial space were affected during the different exercises. That kind of knowledge may contribute to the development of better training regimens [61].

## Conclusion

In this randomised controlled trial, we found no significant difference between a comprehensive supervised training regimen including heavy training principles, and a home-based training program.

## Data Availability

All data and materials, as well as software applications, support our claims and comply with filed standards. The datasets used and/or analysed during the current study are available from the corresponding author on reasonable request.

## Abbreviations

CS: Constant score
HSRT: Heavy slow resistance training
HTR: Home-based regimen
LOCF: Last observation carried forward
LT: Lower trapezius
MT: Middle trapezius
ROM: Range of motion
SA: Serratus anterior
SIS: Subacromial impingement syndrome
SRQ: Shoulder rating questionnaire
STR: Supervised training regimen
UT: Upper trapezius
VAS: Visual analogue scale
